# A Systematic Review of Clinical Applications of Anti-CD20 Radioimmunotherapy for Lymphoma

**DOI:** 10.1093/oncolo/oyad333

**Published:** 2024-01-11

**Authors:** Michael Durando, Ajay K Gopal, Joseph Tuscano, Daniel Persky

**Affiliations:** Division of Hematology and Oncology, University of Arizona School of Medicine, Tucson, AZ, USA; Fargo VA Health Care System, Fargo, ND, USA; Department of Internal Medicine, Division of Hematology and Oncolog, University of Washington School of Medicine, Seattle, WA, USA; Fred Hutchinson Cancer Research Center, Seattle, WA, USA; Division of Malignant Hematology/Cellular Therapy and Transplantation, University of California Davis School of Medicine, Sacramento, CA, USA; Division of Hematology and Oncology, University of Arizona School of Medicine, Tucson, AZ, USA; University of Arizona Cancer Center, Tucson, AZ, USA

**Keywords:** radioimmunotherapy, ibritumomab tiuxetan, efficacy, safety

## Abstract

**Purpose:**

The clinical efficacy of anti-CD20 radioimmunotherapy (RIT) is due to a combination of extracellular mechanisms involving immune-mediated cytotoxicity, and intracellular mechanisms related to inhibition of CD20 signaling and DNA damage from ionizing radiation. In 2002, the first RIT was approved by the U.S. Food and Drug Administration for the treatment of patients with indolent B-cell follicular non-Hodgkin lymphoma (NHL). The 2 approved agents, 90 Y-ibritumomab tiuxetan (90Y-IT, Zevalin, Acrotech Biopharma) and 131 I-tositumomab (131-IT, Bexxar, GlaxoSmithKline) both target CD20. The aim of this study was to review the clinical applications and supporting clinical trial data of anti-CD20 RIT for lymphoma.

**Methods:**

A review of published articles and abstracts on the clinical efficacy and safety of 90Y-IT and iodine I 131 tositumomab was performed.

**Results:**

The clinical efficacy and safety of anti-CD20 RIT have been demonstrated in numerous clinical trials and case series. Agents have produced significant responses in patients with follicular NHLs and in off-label applications. Importantly, RIT has demonstrated promising findings in high-risk lymphomas and heavily pretreated and refractory patient populations. Associated toxicity profiles are noted as tolerable, acceptable, and most often reversible.

**Conclusions:**

In the 2 decades since its approval, anti-CD20 RIT continues to demonstrate efficacy, particularly with a proportion of patients maintaining long-term remissions. The combination of prolonged efficacy, tolerability, and treatment convenience makes RIT a reasonable alternative to other systemic therapies. It is recommended that further research on RIT should focus on biomarkers of long-term response, pretargeting, and sequencing of RIT in the treatment course.

## Teaser

Data supporting the use of anti-CD20 radioimmunotherapy in lymphoma are summarized, with a particular focus on 90 Y-ibritumomab tiuxetan. This update may serve as an evidence-based resource for practicing clinicians, planning of future research, and health-related policy.

**Implications for Practice**

New data regarding the use of radioimmunotherapy (RIT) in oncology have been published over the past 5 years. More specifically, data supporting the use of ibritumomab tiuxetan (Zevalin), a monoclonal antibody RIT and one of the first targeted cytotoxic agents, which has been somewhat overlooked in recent years despite excellent data and a good safety record. This review summarizes the most recent evidence, as well as provides a critical appraisal of these data and its clinical implications. This update may serve as an evidence-based resource for practicing clinicians, planning of future research, and health-related policy.

## Introduction

Approved in 1997, Rituximab was the first monoclonal antibody approved for cancer treatment. By binding selectively to CD20, a transmembrane protein highly expressed on B-lymphocytes, rituximab causes highly targeted, direct, and cell-mediated cytotoxicity, becoming the foundation of many B-cell lymphoma treatment regimens. Adding cytotoxic payloads, such as radionuclides, to monoclonal antibodies led to the development of radioimmunotherapy (RIT), which is the focus of this review.

RIT combines highly specific localization of monoclonal antibody technology with cytotoxicity of ionizing radiation to deliver selective, cytotoxic therapy. The ideal antigen target is uniformly and selectively expressed on malignant cells, such that the monoclonal antibody (“immunotherapy”) binds to tumor-specific antigens, after which the radionuclide (“radiotherapy”) releases ionizing beta particles within a finite radius, causing single- and double-stranded DNA breaks in nearby cells and resultant cellular death.

In February 2002, the first RIT was approved by the U.S. Food and Drug Administration (FDA) for the treatment of patients with indolent B-cell follicular non-Hodgkin lymphoma (NHL). The 2 approved agents, 90 Y-ibritumomab tiuxetan (90Y-IT, Zevalin, Acrotech Biopharma) and 131 I-tositumomab (131-IT, Bexxar, GlaxoSmithKline) both target CD20.

To review the evidence of RIT in lymphoma, we searched electronic databases, including PubMed, the Cochrane Library, Web of Science, and MEDLINE for original studies, case reports, and case series from 2015 to 2023. All authors have expertise in the clinical and preclinical subject matter and reviewed papers identified in the search. All authors came to a consensus on those publications to be included in the review. Only English-language publications were included (Table 1).

**Table 1.** Reviewed clinical studies of outcomes of RIT.

Author (year of publication)	Study design	Condition treated (n)	Outcomes	Study conclusion
Morschauser (2008)^5^	Randomized, open-label, phase III	Previously untreated advanced follicular lymphoma, achieved PR or CR to first-line induction therapy (414 = 208 90Y-IT consolidation, 206 control)	Following consolidation with 90Y-IT, 77% PR to CR/unconfirmed CR (CRu) conversion rate, final CR rate of 87% 53% for control	Consolidation of first remission with 90Y-IT in advanced-stage follicular lymphoma is highly effective with no unexpected toxicities, prolonging PFS by 2 years and resulting in high PR-to-CR conversion rates regardless of type of first-line induction treatment
Morschauser (2013)^6^	Long-term follow-up to 2008 study	Previously untreated advanced follicular lymphoma, achieved PR or CR to first-line induction therapy (409 = 207 90Y-IT, 202 control)	Estimated 8-year overall PFS 41%, 22% for control patients in CR/CRu after induction, 8-year PFS with 90Y-IT was 48%, 32% for controls	90Y-IT consolidation after achieving PR or CR/CRu to induction confers 3-year benefit in median PFS with durable 19% PFS advantage at 8 years and improves TTNT by 5.1 years for patients with advanced FL
Horning (2005)^7^	Multi-center, single-arm, prospective phase II	Low grade or transformed low- grade or grade 3 lymphoma whose disease had not responded to or had progressed after at least 4 doses of rituximab therapy (40)	OR 68%, median DoR 16 months CR 33%, median DoR not reached	131-IT is effective in CD20-positive lymphoma progressive after rituximab, with a 65% OR rate and median PFS of 24.5 months for responders. Patients with follicular grade 1 or 2 histology and tumors ≤7 cm achieved very high OR and CR rates, with 48% PFS at 3 years
Lansigan (2019)^9^	Prospective, multicenter, phase II	Untreated follicular lymphoma (39)	OR 95% CR/Cru 77% increasing to 81% after RIT consolidation. At median follow-up of 45 months, OS 96%, PFS 71%	Four cycles of BR followed by consolidation with 90YIT achieve high response rates that are durable. Consolidation with 90Y-IT results in a high conversion rate of PR to CR/CRu. A short course of BR followed by 90Y-IT is a safe and effective regimen for frontline treatment of FL
Miura (2021)^12^	Prospective	Follicular lymphoma (22)	OR 88% At median follow-up of 46.8 months, 2-year PFS after consolidation 59%	90Y-IT consolidation after BR re-induction conferred a durable remission for patients with relapsed FL
Casadei (2016)^13^	Long-term follow-up of multicenter, nonrandomized, phase II	Untreated patients with intermediate/high-risk follicular lymphoma (55)	Median follow-up of 84 months, 39% of CR patients had relapsed, resulting in an estimated long-term disease-free survival of 63%. 7-year OS 73%. Secondary AML 7.3%, median 42 months following RIT	Data represented the first evidence of a real role even in the long period of 90Y-IT after a fludarabine-containing regimen plus rituximab in the treatment of high-risk follicular lymphoma
Samaniego (2021)^15^	Single-institution single-arm phase II	Advanced follicular lymphoma (47)	Following R-FND, CR and PR were 91% and 8.5%, respectively. After 90Y-IT consolidation, CR increased to 97%. 10-year PFS was 49%	This treatment approach is most appropriate in FLIPI-based high-risk patients whose outlook with standard therapy is inadequate
Puvvada (2018)^16^	Prospective case series	Follicular lymphoma (28)	ORR 72% CR 45% median follow-up 73 months, 1-year PFS 38%, median PFS 10 months, median OS not reached	Primary endpoint of 1-year PFS of 67.3% not reacehd. Reasons for this could include low accrual, high-risk disease, and inadequate debulking provided by 2 cycles of ESHAP. However, this protocol was associated with tolerable toxicity, high ORR, and high OS
Moustafa (2019)^17^	Retrospective case series	Follicular lymphoma (137)	Median follow-up from 90Y-IT of 10.2 years, 69% were alive. Median PFS was 2.5 years and time to next therapy was 3.6 years. Significantly higher median PFS in previously untreated patients, 4.1 years, vs 2.2 years for the relapsed/refractory low-grade	90Y-IT is an effective single-agent regimen for low-grade FL. The response and survival data in this large real-world cohort are superior to the pivotal trials of this agent conducted nearly 20 years ago
Moustafa (2022)^18^	Long-term Follow-up	Marginal zone NHL relapsed (21) 52% previously-untreated	ORR 91%, 43% relapsed and 81% were alive at last data collection. CR 81%, 65% remain in CR at median follow-up of 5.7 years	90Y-IT is efficacious and well tolerated in patients with previously untreated as well as relapsed MZL. As expected it appears to be more efficacious in previously untreated patients
López-Guillermo (2022)^19^	Randomized phase II compare consolidation vs maintenance	Follicular lymphoma (146) in PR or CR/CR[u] after R-CHOP	Median follow-up 10.55 years, 10-year PFS of 50% (90Y-IT) vs 56% (maintenance), 10-year OS 78% (90Y-IT) vs 84.5% (maintenance)	In FL patients responding to R-CHOP, no significant differences were found between consolidation and maintenance
Rieger (2022)^20^	Long-term follow-up	Follicular lymphoma (59)	Median follow-up 9.6 years, median PFS 3.6 years, 8-year PFS was 38.3%. Median OS was not reached, 8-year OS 69.2%	90Y-IT as first-line treatment demonstrates a favorable safety profile and long-term clinical activity in a substantial proportion of FL patients
Lossos (2020)^21^	Single-institution single-arm phase II	Marginal Zone NHL (17)	ORR at 12 weeks 88.4%, CR 53%, CRu 6% and PR 29.4%. Median DoR 4.2 years. Median follow-up 8.3 years, median PFS 4.3 years. PFS at 5 and 8 years, 41.2% and 34.3% and OS at 5 and 8 years, 76.4% and 68.6%	Overall, 90Y-IT was well tolerated and led to long-term responses and PFS rates
Lolli (2020)^22^	Single-institution single-arm phase II	Mucosa-associated lymphoid tissue (MALT) subtype of Marginal Zone NHL (16)	3 months post treatment ORR 94%, CR 62.5%, and PR 31.3%. Median 2 year follow-up, 4 patients relapsed, and one death occurred due to disease progression. No secondary malignancies occurred. PFS at 71.9 months 42.6%, median reached at 37.3 months	90Y-IT represents a highly effective and tolerable treatment option for MALT lymphoma, in a setting where currently there are no standard treatments and the therapy options are limited, especially in R/R patients
Zinzani (2010)^24^	Phase II	Untreated diffuse large B-cell lymphoma (DLBCL) high-risk elderly (55)	ORR 80%, 73% CR and 7% PR. Half of those patients that did not achieve a CR with CHOP, improved remission status with 90Y-IT. Median follow-up 18 months, 2-year PFS estimated 85%, 2-year OS 86%	This study evaluated the feasibility, efficacy, and safety of a short-course R-CHOP21 regimen followed by 90Y-IT in high-risk elderly DLBCL patients
Witzig (2015)^25^	Phase II	Early stage (I/II) DLBCL (53)	Following RCHOP, 79% in CR/Cru, and 48 proceeded to RIT, which resulted in metabolic CR of 89%, without requiring IFRT. Median follow-up 5.9 years, 13% progressed and 8% died. At 5 years, 78% remained in remission and 94% were alive	Chemoimmunotherapy and RIT are an active regimens for early-stage DLBCL patients. Eighty-nine percent of patients achieved functional CR without the requirement of IFRT
Morschauser (2007)^26^	Prospective, multicenter, nonrandomized phase II	Elderly population in first relapse of primary refractory diffuse large B-cell lymphoma ineligible for transplant	ORR 52%, median PFS 5.9 months for previously chemotherapy treated, whom induction therapy failed ORR 53% median PFS 3.5 months for previously treated with chemotherapy, and relapsed after CR ORR 19% median PFS 1.6 months for previously treated with chemotherapy plus rituximab	90Y-IT is active in patients with relapsed and refractory DLBCL. Nonhematologic adverse events were mild-to-moderate
Arnason (2015)^27^	Phase II	DLBCL not candidates for transplant (25)	ORR of 36%, median EFS of 2.5 months and OS of 8.1 months	The ORR of 36% with 90Y-IT as salvage therapy for DLBCL while inferior to more aggressive regimens is significant with acceptable toxicity. For a subset of patients not candidates for salvage with autologous transplant, this treatment strategy can produce a durable, long-lasting remission
Persky (2015)^28^	Phase II	Early-stage aggressive B-cell NHL (43)	PFS estimate of 89% at 2 years, 82% at 5 years, and 75% at 7 years. The OS estimate was 91% at 2 years, 87% at 5 years, and 82% at 7 years	Patients with high-risk LD-NHL treated with 3 cycles of CHOP plus IFRT followed by 90Y-IT consolidation had outcomes that compare favorably to our historical experience
Persky (2020)^29^	Phase II	Early stage DLBCL (132, 14 iPET positive)	67% converted PR to CR and 33% had PR, overall CR 92% and PR 4% (with 4% unevaluable). Median follow-up 4.5 years, only 6 patients progressed and 3 died from lymphoma. 5-year PFS estimate 87% and OS estimate 89%, with iPET-positive and iPET-negative patients having similar outcomes	With PET-directed therapy, 89% of the patients with a negative iPET received R-CHOP × 4, and only 11% had a positive iPET and required radiation, with both groups having excellent outcomes
Chow (2022)^30^	Phase II -megadose 90Y-IT, fludarabine, and low-dose total body irradiation + HLA-matched allo-HCT	CD20 + BCL patients (20)	Estimated 1- and 5-year PFS 55% and 50%, median PFS 1.57 years. Estimated 1- and 5-year OS 80% and 63%, median OS 6.45 years	Megadose 90Y-IT was feasible, safe, and effective in treating aggressive BCL, exceeding the prespecified endpoint, nonhematologic toxicities comparable to those of standard reduced-intensity conditioning regimens
Ciochetti (2018)^31^	Retrospective	Aggressive NHL, relapsed or refractory to a rituximab-containing chemotherapy (37)	90Y-IT 59% CR, 27% PR. Median follow-up 61 months, 3-year PFS 61% and OS 61%. Fifteen patients died, 12 of lymphoma	In relapsed/refractory high-risk NHL, Z-BEAM + ASCT is able to achieve a good ORR. Three-year PFS is promising for early relapsed patients but is not satisfactory for those with refractory disease
Cabrero (2017)^33^	Phase II	High risk NHL (18)	Nonrelapse mortality at 1 year 28%. Median follow-up 46 months and estimated 1-year PFS 50%, and 4-year OS and PFS both 44%	Y90-IT as a component of RIC for AlloSCT is feasible in patients with high-risk B cell lymphoma
Bouabdallah (2015)^34^	Phase II	Diffuse Large B-Cell Lymphoma/MCL (31)	Median follow-up 32 months, 2-year EFS and OS both 80%	For chemosensitive advanced high-risk B-cell lymphoma, the addition of 90Y-IT to a RIC regimen based on fludarabine, busulfan, and antithymocyte globulin followed by allogeneic transplant is safe and highly effective
Othman(2019)^35^		90Y-IT in the total lymphoid irradiation and anti-thymocyte globulin conditioning regimen for nonmyeloablative alloSCT (16)	The conditioning regimen including 90Y-IT was well tolerated and had a conversion rate and 2-year EFS of 82% and 71%, respectively	The authors considered these to be promising findings, considering that this patient population was heavily pretreated and mostly refractory
Puronen (2018)^36^	Long-term follow-up	Indolent B NHL (40)	At 9 years, OR 40% PFS 27.5%	90Y-IT–based allografting represents a viable option in patients with indolent histologies
Hohlich (2020)^37^	Registry analysis	Mantle cell lymphoma (90)	67% and 22% achieved CR and PR, respectively. Median PFS and OS were 2.1 and 4.1 years, respectively, at median follow-up of 5.5 years	90Y-IT was most often used as consolidation after first- and second-line chemotherapy and may improve the results achieved using chemoimmunotherapy alone. However, the results are less encouraging compared to treatment with small molecules such as ibrutinib
Witzig (2002)^47^	Prospective	Relapsed follicular non-Hodgkin’s lymphoma (54)	OR 74% CR 15%	90Y-IT is effective in rituximab-refractory patients. The only significant toxicity is hematologic
Witzig (2002)^48^	Randomized, controlled, phase III	Relapsed or refractory low-grade or follicular non-Hodgkin’s lymphoma or transformed B-cell non-Hodgkin’s lymphoma (143=73 90Y-IT + 70 Rituximab)	90Y-IT: OR 80%, CR 30%, Rituximab: OR 56%, CR 16% Durable responses (≥6 months) 90Y-IT: 64%; Rituximab: 47%	Radioimmunotherapy with 90Y-IT is well tolerated and produces statistically and clinically significant higher ORR and CR compared with rituximab alone

### 90 Y-Ibritumomab Tiuxetan and 131 I-Tositumomab

90Y-IT (Zevalin) was the first RIT to receive FDA approval in hematologic malignancies, approved in February 2002 for the treatment of CD20-positive, relapsed or refractory (R/R), low-grade, or follicular B-cell NHL, expanded in 2009 to include patients with previously untreated follicular NHL who had achieved a partial or complete response to first-line therapy. 90Y-IT is composed of 3 main components: a CD20-targeting antibody (ibritumomab), a metal ion chelator and linker (tiuxetan), and the radionuclide Yttrium-90 (90Y). The mechanism of action of 90Y-IT has been well described, previously.^1-4^

The First-Line Indolent Trial (FIT) was a prospective, randomized, open-label, phase III international trial comparing no further treatment to consolidation therapy with 90Y-IT in patients with previously untreated advanced FL who achieved a partial response (PR) or complete response (CR) to first-line induction therapy.^5^ First-line induction included chlorambucil, CVP/COP (cyclophosphamide, vincristine, and prednisone), CHOP (cyclophosphamide, doxorubicin, vincristine, and prednisone), and fludarabine combinations or rituximab combination regimens. Following consolidation with 90Y-IT, patients demonstrated a 77% PR to CR/unconfirmed CR (CRu) conversion rate, with a final CR rate of 87%, compared to 53% for controls. At a median 3.5 years of follow-up, the median progression-free survival (PFS) was significantly prolonged with 90Y-IT. In 2013, FIT Investigators published updated results after median follow-up of 7.3 years.^6^ Of 409 patients available for analysis, the 207 who received 90Y-IT had an 8-year overall PFS of 41%, compared to 22% for controls. For patients in CR/CRu after induction, 8-year PFS with 90Y-IT was 48%, versus 32% for controls. For 90Y-IT consolidation, median PFS was 4.1 years and median time to next treatment was 8.1 years, compared to 3 years for control patients with no significant difference in overall survival (OS) between treatment groups.^6^

131-IT (Bexxar) a murine anti-CD20 IgG2 monoclonal antibody, tositumomab, directly labeled with iodine‐131, a dual beta/gamma emitter. 131-IT was approved by the FDA in June 2003 based on results from a multi-center, single-arm study of 40 subjects with low-grade or transformed low-grade or grade 3 lymphoma whose disease had not responded to or had progressed after at least 4 doses of rituximab therapy.^7^ The OR was 68%, with a median duration of response (DoR) of 16 months, CR was 33% and the median DoR was not reached.^7^ In 2014, GlaxoSmithKline halted the manufacturing of 131-IT due to low utilization. Since then, the long-term follow-up of SWOG0016, comparing R-CHOP to CHOP followed by 131-IT has been published, indicating an improved 10-year PFS in the RIT group compared to R-CHOP (56% vs 42%) but similar OS (75% vs 81%) between arms.^8^

### Consolidation Strategies and Long-Term Outcomes

As RIT has the potential to eliminate residual disease, several studies, in addition to the FIT trial, have used it for consolidation. Fol-BRITe was a prospective multicenter trial that evaluated the response rate, PFS, and tolerability of BR followed by consolidation with 90Y-IT in patients with untreated FL.^9^ The study included 39 chemotherapy-naïve patients with stages II-IV disease treated with rituximab, 375 mg/m^2^, followed by four 28-day cycles of rituximab 375 mg/m^2^ on day 1 and bendamustine 90 mg/m^2^ on days 1 and 2. Patients were eligible for consolidation with 90Y-IT, 6-12 weeks later, if they obtained a PR after induction and had adequate count recovery and bone marrow infiltration <25%. The Fol-BRITe investigators concluded that a short course of BR followed by RIT was safe and effective for front-line treatment of FL, noting a 95% ORR, and a CR/CRu rate of 77%, which increased to 81% after RIT consolidation. At a median follow-up of 45 months, OS and PFS were 96% and 71%, respectively.^9^ Although this study included a relatively small number of patients, the high ORR and proportion obtaining CR/CRu, compares favorably to the BRIGHT and StiL studies of 6 cycles of rituximab-bendamustine, demonstrating a CR of 31% and 40%, respectively.^10,11^

Miura recently reported a series of patients with relapsed FL (*n* = 22) who received consolidation with 90Y-IT, with median follow-up of 46.8 months. Estimated 2-year PFS after consolidation was 59%, leading authors to suggest that consolidation with 90Y-IT may be a novel therapeutic option for these patients with relapsed FL.^12^

The role of 90Y-IT in consolidation for high-risk FL was demonstrated by Casadei, evaluating long-term efficacy and toxicity of sequential treatment with 4 cycles of fludarabine, mitoxantrone, and rituximab (R-FM) followed by 90Y-IT as front-line therapy for untreated patients with intermediate/high-risk FL (*n* = 55).^13^ At a median follow-up of 84 months, 39% of patients with CR had relapsed, resulting in an estimated long-term disease-free survival of 63% and a 7-year OS of 73%. Comparatively, standard R-FM, after median follow-up of 4 years, was reported to have a 3-year PFS of 47%, and 3-year OS of 66%.^14^

Samaniego reported that following treatment with treated with R-FND (rituximab, fludarabine, mitoxantrone, and dexamethasone), their group of 47 patients with advanced FL achieved CR and PR rates of 91% and 8.5%, respectively. After consolidation with 90Y-IT, the CR rate increased to 97% and the 10-year PFS rate was 49%.^15^

Puvvada also hypothesized that RIT eliminated minimal residual disease after cytoreduction in FL, thus facilitating durable complete remissions.^16^ Twenty-eight patients with FL received 2 cycles of ESHAP (etoposide, methylprednisolone, cytarabine, and cisplatin) every 28 days, followed by 90Y-IT 4 to 6 weeks later if there had been no disease progression and <25% bone marrow involvement. The ORR was 72%, with 45% achieving CR. With a median follow-up of 73 months, 1-year PFS was 38%, and median PFS was 10 months, but median OS was not reached. The authors concluded that, although the primary endpoint of the study was not reached (PFS 67.3%), the treatment protocol was associated with tolerable toxicity, high ORR, and high OS and future studies should optimize debulking and focus on patients with high-risk FL.^16^

At ASH 2019, Moustafa presented the long-term outcomes of 137 FL patients treated with 90Y-IT.^17^ At a median follow-up from 90Y-IT of 10.2 years, 69% were alive. Median PFS was 2.5 years and time to next therapy, 3.6 years. A significantly higher median PFS was observed in previously untreated patients, 4.1 years, compared to 2.2 years for the R/R low-grade group. Additionally, ~25%, both in first-line and R/R setting, maintained a CR to RIT at up to 13 years, highlighting both long-term efficacy of RIT and the need for further prospective studies.^17^ The same authors recently published their long-term real-world outcomes of 41 previously untreated patients with FL followed for a mean 5.3 years demonstrating durable long-term survival.^18^ The ORR was 100% with CR of 95% and continuous CR observed in 54% of those patients with >2 years of follow-up. Long-term CR (>7 years) was seen in 25% of patients.^18^

Lopez recently reported long-term results (median 10.55 years) comparing consolidation with a single dose of 90Y-IT to 2-years of maintenance with rituximab in 146 newly diagnosed patients with FL responding to R-CHOP.^19^ Fifty-three progressed with a 10-year PFS of 50% for those randomized to 90Y-IT, compared to 56% for rituximab.^19^

Rieger reported long-term (median 9.6 years) follow-up of 59 patients with FL who received 90Y-IT as first-line treatment.^20^ They reported a median PFS of 3.6 years, an 8-year PFS of 38.3%, and 8-year OS of 69.2% with a median OS that was not reached.^20^ Patients ≥65 years or those with disease progression within 24 months of treatment were associated with significantly shorter OS.^20^

### Radioimmunotherapy in Indolent Nonfollicular NHL

RIT has demonstrated clinical efficacy in other CD20-expressing B-cell NHLs. Marginal zone lymphomas (MZL) is recognized as being sensitive to radiation, therefore a more targeted form of radiation with systemic distribution could be an effective therapy. Lossos conducted a phase II study of 90Y-IT as a front-line treatment for 17 patients with MZL, 76.5% advanced-stage and 41% with bulky disease.^21^ 90Y-IT was well tolerated with an ORR of 88.4% at 12 weeks including a CR in 53%, CRu in 6%, and PR in 29.4%. After a median follow-up of 8.3 years, median PFS was 4.3 years, and median OS was not reached. Two-, 5-, and 8-year PFS were 58.8%, 41.2%, and 34.3%, respectively. The OS rates at 2, 5, and 8 years were 94.1%, 76.4%, and 68.8%, respectively.^21^

Lolli evaluated 90Y-IT treatment in 16 patients with newly diagnosed or R/R MALT subtype of MZL.^22^ At 3 months post-treatment, the ORR was 94%, with a CR of 62.5%, and PR of 31.3%. At a median 2-year follow-up, 4 patients relapsed, and one death occurred due to disease progression. No secondary malignancies occurred. Long-term follow-up revealed that 42% of patients had a PFS of 72 months, with a median PFS of 37 months among all patients.^22^

Long-term outcomes of 90Y-IT treatment for previously untreated and relapsed MZL were recently reported by Moustafa.^23^ Their study included 21 MZL patients treated with 90Y-IT, 52% previously untreated, who were evaluated after a median follow-up of 8.5 years. Treatment was well tolerated, the ORR was 91%, 43% relapsed, and 81% were alive at last data collection. The CR rate was 81% and 65% remained in CR at a median follow-up of 5.7 years. 90Y-IT was more efficacious in previously untreated patients and long-term complete remission (>5 years) was observed in 52.%. The same group recently published their outcomes of 10 previously untreated patients with MZL followed for a mean 5.3 years that showed durable long-term survival.^23^ The ORR was 100% with CR of 90% and continuous CR observed in 59% of those patients with>2 years of follow-up. Long-term CR (>7 years) was seen in 25% of patients.^23^

### Radioimmunotherapy in Aggressive NHL (DLBCL)

Zinzani evaluated efficacy and safety of 90Y-IT following R-CHOP in untreated DLBCL, a potentially curable and the most common NHL.^24^ The study population included 55 high-risk older (≥60 years) patients, of which 48 underwent RIT. The ORR was 80%, which also included 73% CR and 7% PR. Half of those patients who did not achieve a CR with R-CHOP improved their remission status with 90Y-IT. With a median follow-up of 18 months, 2-year PFS was estimated to be 85%, with a 2-year OS of 86%.^24^

The ECOG3402 study investigators evaluated a sequential treatment approach for early stage (I/II) DLBCL, including 4-6 cycles of R-CHOP, followed by RIT, reserving 30 Gy involved field radiation therapy (IFRT) for those not in metabolic CR positron emission tomography (PET negative) after RIT. A phase II trial of 53 patients, 79% were in CR/CRu following R-CHOP, and 48 proceeded to RIT, resulting in a metabolic CR rate of 89%, with omission of IFRT. With a median follow-up of 5.9 years, 13% progressed and 8% died. At 5 years, 78% remained in remission and 94% were alive.^25^

Earlier, Morschauser et al had conducted a prospective, multicenter trial evaluating 90Y-IT in older (≥60 years) patients in first relapse of primary refractory DLBCL, ineligible for transplant. The ORR was 52% in patients who were primary refractory to chemotherapy, 53% for relapsed patients who relapsed after chemotherapy and 19% for those previously treated with chemotherapy plus rituximab. Median PFS was 5.9, 3.5, and 1.6 months, respectively.^26^ Arnason investigated 90Y-IT in 25 patients with DLBCL that were not candidates for transplant; they reported an ORR of 36%, with a median event-free survival (EFS) of 2.5 months, an OS of 8.1 months, and grade 3 nonhematologic toxicities in 36% of patients.^27^

The SWOG S0313 study evaluated the addition of 90Y-IT consolidation to 3 cycles of standard CHOP plus IFRT in 43 patients with early-stage aggressive B-cell NHL.^28^ The treatment regimen included CHOP on days 1, 22, and 43, followed 3 weeks later by 40-50 Gy of IFRT. 90Y-IT was initiated 3-6 weeks later. 90Y-IT–treated patients had comparatively favorable outcomes, with a PFS estimate of 89% at 2 years, 82% at 5 years, and 75% at 7 years. The OS estimate was 91% at 2 years, 87% at 5 years, and 82% at 7 years.^28^ Based on these findings, SWOG designed S1001, the largest U.S. study of early stage DLBCL, which was a PET-directed study to tailor therapy after 3 cycles of R-CHOP. The study included 132 patients, of which only 14 were determined to be truly iPET-pos (interim PET-positive). Two patients refused radiation, and 12 received IFRT-90Y-IT; of these, 67% converted from PR to CR and 33% had PR, for an overall CR of 92% and PR of 4%. With median follow-up of 4.5 years, 6 progressed and 3 died from lymphoma. The 5-year PFS and OS estimates were 82% and 87%, respectively, with iPET-positive and iPET-negative patients having similar outcomes.^29^

More recently, Chow evaluated a high dose (median 113.6 mCi) of 90Y-IT prior to allo-HCT in 20 patients with R/R aggressive BCL.^30^ Estimated 1- and 5-year PFS was 55% and 50% with median PFS of 1.57 years and estimated 1- and 5-year OS was 80% and 63%, median OS of 6.45 years. Although 80% experienced ≥grade 3 toxicities, nonrelapse mortality was 10% at 1 year.^30^ Results exceeded the prespecified endpoint, and nonhematologic toxicities were comparable to standard dose regimens.

### Radioimmunotherapy in Transplant Conditioning Regimens

In recent years, RIT has been added to autologous stem cell transplantation (ASCT) conditioning regimens in aggressive NHL. Ciochetto conducted a retrospective analysis of patients with aggressive NHL, R/R to rituximab-containing chemotherapy, who received 90Y-IT followed by the high-dose conditioning regimen BEAM (carmustine, etoposide, citarabine, and melphalan) and ASCT or matched controls who received only BEAM before ASCT.^31^ The 90Y-IT group had a CR and PR of 59% and 27%, respectively, and at 61 months, the 3-year PFS and OS were both 61%. Within the study period, 15 died, 12 of lymphoma. A numerically, but nonstatistically significant, higher 3-year PFS was observed for those receiving 90Y-IT, compared to the control group.^31^ An earlier study comparing BEAM with iodine-131 tositumomab/BEAM with ASCT for chemotherapy-sensitive relapsed DLBCL produced similar 2-year PFS and OS rates.^32^

A phase II GELTAMO trial included 90Y-IT as part of a reduced-intensity conditioning for allogeneic stem cell transplantation (AlloSCT) in high-risk NHL.^33^ Of 18 evaluable patients, at time of transplantation, 11 (61%) had active disease, 33% in PR, 39% in CR, and 28% had stable disease.

Nonrelapse mortality at 1 year was 28%. Median follow-up was 46 months, estimated 1-year PFS was 50%, and 4-year OS and PFS were both 44%. Although the addition of 90Y-IT was feasible in this patient group, the authors concluded that larger studies would be needed to more precisely determine its contribution to reduced-intensity conditioning for alloSCT.^34^ Bouabdallah incorporated 90Y-IT into a fludarabine-based RIC and prospectively evaluated 31 patients with advanced lymphoma from 5 institutions.^34^ At median follow-up of 32 months, the 2-year EFS and OS were both 80%, leading the authors to conclude that for chemosensitive advanced high-risk B-cell lymphoma, adding 90Y-IT to the regimen, followed by alloSCT was safe and highly effective.^34^

More recently, Cardenas reported preliminary findings of adding 90Y-IT to the total lymphoid irradiation and anti-thymocyte globulin conditioning regimen for nonmyeloablative alloSCT.^35^ The conditioning regimen, including 90Y-IT, was well tolerated and had a conversion rate and 2-year EFS of 82% and 71%, respectively. The authors considered these to be promising findings, considering that this population was heavily pretreated and mostly refractory.^35^

In 2018, Puronen published long-term outcomes of patients with indolent B-NHL that received 90Y-IT-based allografting, specifically emphasizing those that achieved early remission.^36^ Forty were alive at the median follow-up of 9 years, with a 5-year OS and PFS of 40% and 27.5%, respectively.^36^

90Y-IT is not currently approved for mantle cell lymphoma (MCL), but Hohlich recently published a study assessing the long-term efficacy of RIT (90Y-IT) in patients with MCL.^37^ They assessed data from 90 patients registered in the web-based RIT Network. Half received 90Y-IT first-line and the remaining half received it in the R/R setting, of those, 98% received 90Y-IT as consolidation after chemoimmunotherapy in first line and 53% after relapse. As a first-line treatment, 67% and 22% of patients achieved CR and PR, respectively, and median PFS and OS were 2.1 and 4.1 years, respectively, at a median follow-up of 5.5 years.^37^

Collectively, these studies demonstrate that RIT-based strategies provide robust and durable responses in a variety of B-cell lymphoma subtypes, regardless of the lymphoma risk profile, histology, or line of therapy when it is used (eg primary therapy, consolidation, or conditioning).

### Safety and Toxicity

The most common toxicity associated with RIT is reversible myelosuppression, with recovery within 2-4 weeks. Approximately 20% of patients require supportive therapy, including growth factor, platelets, and blood transfusion.^38^ In the early clinical studies of 90Y-IT, 30% experienced grade 4 neutropenia, 10% thrombocytopenia, and anemia occurred in 4%.^39^ Increased hematologic toxicity has been associated with the number of previous treatment regimens, degree of bone marrow involvement and prior fludarabine treatment.

Although the efficacy of RIT is well established, a limiting factor to utilization may be related to concerns related to radiation-induced myelodysplastic syndromes (MDS), progressing to AML. It has been estimated that up to 10% of patients with NHL treated with either conventional-dose chemotherapy or high-dose therapy and ASCT may develop MDS/AML within 10 years of primary therapy.^40^ Cited as a likely consequence of diffuse irradiation of the bone marrow, a pooled analysis of MDS and AML in 746 patients with NHL treated with 90Y-IT in registration and compassionate-use trials between 1996 and 2002 found that 2.5% of patients developed these secondary malignancies at a median follow-up of 4.4 years.^41^ Within the registration trials of Bexxar (131-IT), MDS or AML was reported in 10%, and 3% of patients enrolled in the expanded access program (median follow-up of 39 and 27 months, respectively). The median time to development of MDS/AML was 31 months. Rates of MSD/AML have also been reported as high as 8%-13% in single-institution retrospective series.^42,43^ In the long-term outcome report from FIT, Morschauser et al described an annualized incidence rate of MDS/AML of 0.50% in the 90Y-IT group, compared to 0.07% in controls (*P* = .042).^6^

In 2005, Bennet studied whether systemic radiation associated with RIT compounds the expected incidence of treatment-related MDS/AML in heavily treated patients.^44^ They evaluated MDS/AML incidence in heavily pretreated patients with R/R low-grade NHL prior to RIT (131-IT) administration and compared them to patients who had received RIT as initial therapy for follicular low-grade NHL. Following a median follow-up of 6 years, of 995 previously treated patients, investigators reported 35 cases of MDS/AML, while no case of MDS/AML was reported in the 76 patients receiving RIT as initial therapy.^44^ Within the SWOG S0016 analysis of CHOP plus rituximab or CHOP plus ^131^I-Tositumomab, 5 (1.8%) treated with R-CHOP and 13 (4.9%) who received CHOP-RIT developed AML or MDS.^8^ Although rates of MDS/AML were higher than in other reports, patients receiving CHOP-RIT had significantly better 10-year PFS compared with patients in the R-CHOP arm (56% vs 42%).^8^

Andrade-Campos also sought to address the controversy surrounding an increased risk of secondary neoplasms in patients with FL treated with RIT.^45^ In the single institution report, they assessed 242 patients with FL, 40% treated with RIT, followed for a median of 61 months. There was no difference in incidence of secondary neoplasms related to exposure to RIT, although the authors recommended avoiding its use after fludarabine and other intense cytotoxic regimens.^45^

Most recently, Di et al examined population-based, real-world use of 90Y-IT and the associated incidence of treatment-related MDS/AML.^46^ They found that prior exposure to a purine analog or alkylating agent remained significantly associated with increased risk of MDS/AML. Similar to Bennet, they also observed no MDS/AML in patients who received RIT as first-line treatment.^46^ The authors drew 3 important conclusions: that earlier use of RIT may be preferable when considering MDS/AML risk; that risk among older patients was reassuringly low; and finally, they recommend screening for clonal hematopoiesis, which is strongly associated with MDS/AML after cytotoxic exposure. However, it is important to note that the cytotoxic chemotherapy induction regimens associated with secondary cancers (reference 40, add references 47 (PMID 16520465) and 48 (18698083)) have been largely replaced by immuno- and chemo-immunotherapy regimens, in particular for indolent lymphomas, with which an association with secondary cancers is neither established nor expected.

### Quality of Life

RIT is considered to have a low treatment burden for patients, due to its single-dose administration. It provided a unique treatment option for patients with geographic or demographic barriers to routine treatment requiring frequent visits with intravenous administration. The 2 key approval studies of 90Y-IT included a quality-of-life assessment using the FACT-G survey. These heavily pretreated patients showed a significant improvement in r overall FACT-G total score, as well as their FACT-G subscale scores for physical, social, emotional, and functional well‐being with 90Y‐IT treatment.^47,48^

More recently, Andrade-Campos et al evaluated the health-related quality of life of 37 patients with FL, treated with 90Y-IT outside of a clinical trial setting.^49^ Patients had been followed for ≥5 years and results of the SF-36 health survey were compared to those from the general population in Spain. Compared with the general population, there were clear negative differences in the scores for physical and emotional items, slightly negative for physical function, bodily pain, and mental health, and slightly better scores for the general health, vitality, and emotional items. No apparent differences were found between relapsed and nonrelapsed patients.^49^

Altogether, clinical trials and real world data have consistently shown that RIT provides an excellent balance of tolerability and efficacy that provides robust OS and PFS advantages without significantly compromising quality of life.

### Utilization

Despite the efficacy and manageable safety profile that contributed to FDA approval and inclusion of 90Y-IT within treatment guidelines, its application in clinical practice remains low, especially utilization outside of academic medical centers. Indeed, Bexxar has not been commercially available since 2014, shortly after its manufacturing was discontinued due to declined use.

RIT can only be administered in facilities capable of handling radioactive materials and requires logistical planning for production of the radioactive drug. The coordination of care between referring physicians and facilities administering RIT, including the timing of infusions of rituximab prior to RIT administration can be logistically challenging.

Limited use of 90Y-IT in the community treatment setting has also been attributed to requirements from the Nuclear Regulatory Commission (NRC). The NRC requires oncologists to complete 700 hours of training on the full range of nuclear medicines to be permitted to administer 90Y-IT, a prepackaged RIT, to patients. This training requirement is not feasible for many oncologists, particularly those practicing in the community setting, where ~80% of cancer is treated.^50^

Although concern has been raised about the associated costs of RIT, particularly as it relates to reimbursement, 90Y-IT has one of the lowest cost profiles.^51^ The cost associated with RIT treatment were included in a budget impact modelling analysis of treatment pathways in relapsed low-grade or follicular B-Cell NHL based on NCCN treatment guidelines and 2015 Medicare fee schedule rates. Treatment outcomes were based on the published literature that summarized the ORR, median DOR, and toxicity. Total costs per treatment regimen were based on medical care, drugs administered, and associated adverse effects. The use of 90Y-IT after BR demonstrated a DOR of 7.2 years, and associated treatment sequence costs were $157 712, compared with $224 628 and $145 047 for BR followed by idelalisib and BR followed by rituximab monotherapy, respectively, with a predicted DOR of 6.7 years. The predicted DOR for treatment sequences starting with R-CVP and R-CHOP and followed by 90Y-IT was about 3 years less than BR followed by 90Y-IT at a cost increase of $11 102 and $37 866, respectively. The use of idelalisib after R-CVP or R-CHOP had a predicted DOR of 3.6 years, respectively, with associated costs that were greater than BR followed by 90Y-IT.^51^

### Future Directions

Several studies are currently underway, further assessing RIT in the treatment paradigms of hematologic malignancies. Investigators are evaluating whether 90Y-IT may enhance the efficacy of non-myeloablative alloSCT in patients with R/R NHL with a sub-optimal response to salvage therapy (NCT01811368). Finally, a phase III study is testing whether a single dose of 90Y-IT improves the CR in low tumor burden FL compared to single-agent rituximab (NCT02320292).

The cytotoxic effects of emerging targets in RIT are currently under investigation, and include preclinical and early clinical studies conducted on anti-CD45, anti-CD37, and anti-CD22 targets and their effect on specific lymphoma cell types.

Although 90Y-IT has not been commercially available in recent years, it is anticipated to be available for clinical use by Acrotech Biopharma in 2024.

## Conclusions

New data regarding the use of RIT in lymphoma, particularly as it relates to 90Y-IT (Zevalin), continue to demonstrate efficacy, particularly with a proportion of patients maintaining long-term remissions.

The molecular mechanisms responsible for why a subset of ~25% of patients demonstrate long-term complete responses are poorly understood and throughout this review, no predictive biomarkers for long-term responders were found. The clinical efficacy of RIT is due to a combination of extracellular mechanisms involving immune-mediated cytotoxicity, and intracellular mechanisms related to inhibition of CD20 signaling and DNA damage from ionizing radiation (IR). Further clinical and molecular research, including biomarker studies, will help explain the functional nature of RIT and identify patients who are most likely to benefit from it.

The evidence for RIT causing MDS/AML is confounded by prior chemotherapy use, but a small signal may be present as per FIT trial. Importantly RIT remains one of the simplest regimens in the current indolent lymphoma armamentarium, requiring 2 days for administration, followed by few subsequent laboratory assessments. This impressive efficiency is particularly valuable in times of COVID19 pandemic where minimizing office visits is desirable. Further research on RIT should focus on biomarkers of long-term response, pretargeting, and on sequencing of RIT in the treatment course.

## Data Availability

No new data were generated or analyzed in support of this research.
